# TIM-3, a potential target for sepsis therapy

**DOI:** 10.1016/j.cjtee.2025.08.001

**Published:** 2025-09-06

**Authors:** Shaowen Huang, Xiaofei Huang, Xifeng Feng, Rui Wang, Fengying Liao, Di Liu, Jianhui Sun, Huacai Zhang, Anyong Yu, Ling Zeng

**Affiliations:** a Department of Emergency, Affiliated Hospital of Zunyi Medical University, Zunyi, 563003, Guizhou Province, China; b Department of Critical Care Medicine, Daping Hospital, State Key Laboratory of Trauma, Burns and Combined Injury, Army Medical University, Chongqing, 400042, China

**Keywords:** Sepsis, TIM-3, Immune dysregulation, Immunotherapy, Costimulatory molecule

## Abstract

Immune dysregulation is one of the leading causes of mortality in patients with sepsis. T cell immunoglobulin and mucin domain-containing protein 3 (TIM-3), a negative costimulatory molecule, is pivotal for immune regulation during sepsis. The effects of TIM-3 appear to be bidirectional: in the early stages of sepsis, upregulation of TIM-3 may help attenuate inflammation, whereas its sustained overexpression in later stages and ligand binding promotes immune apoptosis or exhaustion, which suppresses immune responses. Furthermore, TIM-3 synergizes with other immune checkpoint molecules (e.g., programmed cell death receptor-1), exacerbates immunosuppression, and increases the risk of secondary infections. Blocking the TIM-3 signaling pathway can restore immune cell function and represent a novel therapeutic strategy for sepsis. Although TIM-3 holds promise as both a biomarker and a therapeutic target, its mechanisms are complex and may vary across disease stages, which necessitates further research to optimize targeted interventions. Future studies should focus on elucidating the dynamic signaling pathways of TIM-3, developing combination immunotherapies, and conducting clinical trials to validate its safety and efficacy in sepsis treatment.

## 1 Introduction

### 1.1 Background

Sepsis is currently defined as life-threatening organ dysfunction caused by a dysregulated host response to infection.^1^^,^^2^ Epidemiological data indicate that there were approximately 48.9 million sepsis cases worldwide, with the number of sepsis-related deaths reaching 11 million, accounting for nearly 20% of the global all-cause mortality in 2017.^3^ In China, an estimated 4.857 million patients are diagnosed with sepsis annually, of whom 1.265 million progress to severe sepsis or septic shock.^4^ However, despite its prevalence, effective treatments for sepsis are limited.

Despite rapid advancements in life-support technologies within intensive care units, the mortality rate of sepsis has risen continuously in the past decade. This trend shows the inability to rely solely on life-support measures to address the therapeutic challenges associated with sepsis.^5^ The immune system plays an indispensable role in the pathogenesis of sepsis, which suggests that immune-targeted therapies may represent a potential breakthrough in sepsis treatment.

### 1.2 Double-edged sword of the sepsis immune response

The immune system acts as a double-edged sword in sepsis. Appropriate activation of the immune system helps the host promptly eliminate and phagocytose invading pathogens and toxins, whereas excessive activation can damage the host itself.

In the early stages of sepsis, the body rapidly activates the innate immune system to defend against pathogenic invasion, which includes dendritic cells (DCs), macrophages, natural killer cells (NK), and mast cells. These cells phagocytose and neutralize pathogens and toxins, and DCs prime naïve T cells through major histocompatibility complex (MHC)-mediated antigen presentation, eliciting adaptive immune responses. During the development of sepsis, immune cells trigger a cascade of cytokine release, which promotes the formation of a “cytokine storm”.^6^ Excessive immune responses lead to widespread tissue damage that ultimately progresses to organ dysfunction and even failure: this contributes to the first peak of sepsis-related mortality. Although advanced septic care and diagnosis have enabled some patients to survive this initial critical phase, innate immune cells may remain activated. To prevent further organ damage, the body releases anti-inflammatory factors to counterbalance the excessive inflammatory response. This leads to the suppression or apoptosis of adaptive immune cells (in addition to regulatory T cells), resulting in immunosuppression or immunoparalysis,^7^ which is also referred to as late-phase immune hyporesponsiveness following hyperinflammation.^8^ Some studies suggest that the proinflammatory and anti-inflammatory phases may coexist.^9^ However, immunosuppression compromises the immune function of patients who survive the early stages of sepsis, which makes them highly susceptible to secondary infections and results in a second mortality peak.^7^ Even though they have survived the initial phase of sepsis, the risk for secondary infections remains for a considerable period.

Recent studies have indicated that negative costimulatory molecules—such as programmed cell death receptor-1 (PD-1), cytotoxic T lymphocyte antigen-4 (CTLA-4), B and T lymphocyte attenuator (BTLA), lymphocyte activation-gene-3 (LAG-3), CD244, T cell immunoglobulin and mucin domain-containing protein 3 (TIM-3),^10^ and T cell immunoglobulin and immunoreceptor tyrosine-based inhibitory motif (TIGIT)^11^—may play a key role in mediating sepsis-induced immunosuppression. During sepsis, the expression of these molecules on immune cells is significantly upregulated, which regulates immune responses. Thus, these molecules may be potential therapeutic targets for sepsis immunotherapy.

### 1.3 Basic structure and function of TIM-3

TIM-3 was initially identified in 2002 as a surface marker expressed on Th1 cells.^12^^,^^13^ Studies have revealed that TIM-3 is a type I transmembrane protein that comprises an immunoglobulin domain and a mucin-like domain. Its extracellular region consists of an immunoglobulin variable (IgV)-like domain, followed by a transmembrane domain and a C-terminal cytoplasmic tail.^14^ Notably, the cytoplasmic tail contains 6 conserved tyrosine residues, whose phosphorylation plays a critical role in activating intracellular signaling pathways.^11^

TIM-3, a member of the TIM family, is encoded by genes located on human chromosome 5q33.2 and mouse chromosome 11.^13^ As a negative costimulatory molecule (immune checkpoint), TIM-3 plays a crucial role in the regulation of cellular immunity. Emerging evidence has demonstrated that TIM-3 mediates immune regulation in various pathological conditions, including malignancies,^15^^,^^16^ autoimmune diseases,^17^ transplant rejection,^18^^,^^19^ chronic inflammation,^17^ and persistent infections such as hepatitis B virus,^20^ hepatitis C virus,^21^^,^^22^ human immunodeficiency virus (HIV),^23^ and parasitic infections.^24^^,^^25^ Notably, TIM-3 has protective effects on drug-induced acute kidney injury^26^ and contributes to immune tolerance during pregnancy.^27^^,^^28^

Given its extensive involvement in immune regulation, an important question arises: In the context of sepsis-associated immune dysregulation, what is the functional significance of TIM-3? Could TIM-3 represent a potential therapeutic target for sepsis? This review comprehensively examines current research on TIM-3, focusing on its role in sepsis, its underlying mechanisms, and therapeutic potential as a treatment target for this life-threatening condition.

## 2 Role and mechanism of TIM-3 in severe infections and sepsis

TIM-3 mediates immune regulation via pleiotropic mechanisms, notably by modulating the survival‒apoptosis balance in key immune cell populations (T cells, macrophages, DCs, and NK cells) and reshaping their functional dynamics. Its high expression in the immune system serves as a negative regulatory mechanism by helping to prevent excessive inflammatory responses and maintain immune homeostasis. Current research indicates that TIM-3 primarily regulates immune cells by binding to its ligands, such as T cells, activating downstream signaling pathways, suppressing immune cell proliferation, and promoting their apoptosis and exhaustion. This process reduces the secretion of cytokines (such as interferon-gamma (IFN-γ) and tumor necrosis factor-alpha (TNF-α)) and inhibits immune responses.^29^

In the early stages of sepsis, when TIM-3 has not yet bound to its ligands, human leukocyte antigen B-associated transcript 3 (BAT3) remains bound to the cytoplasmic tail of TIM-3. It then recruits phosphorylated lymphocyte-specific tyrosine kinase to enhance T cell receptor (TCR) signaling, which helps sustain T cell activation and facilitates pathogen clearance. However, as the disease progresses, the excessive release of inflammatory cytokines may cause immune-mediated damage to host tissues and organs. To protect cells and organs from hyperinflammatory injury, the expression of negative costimulatory molecules such as TIM-3 increases in immune cells. TIM-3 then binds to ligands such as galectin-9 (Gal-9), high-mobility group Box 1 (HMGB1), phosphatidylserine (PtdSer), and carcinoembryonic antigen-related cell adhesion molecule 1 (CEACAM-1), and this binding triggers the phosphorylation of tyrosine 256 (Y256) and tyrosine 263 (Y263), which leads to the dissociation of BAT3 from TIM-3. Protein tyrosine kinase Fyn (Fyn) is then recruited and binds to the cytoplasmic tail of TIM-3, and initiates TIM-3-mediated inhibitory signaling. This process activates T cell apoptosis, exerts immunosuppressive effects, promotes immune cell exhaustion, and maintains the immune microenvironmental balance.^11^

### 2.1 TIM-3 signaling mechanisms (Fig. 1)

**Fig. 1:**
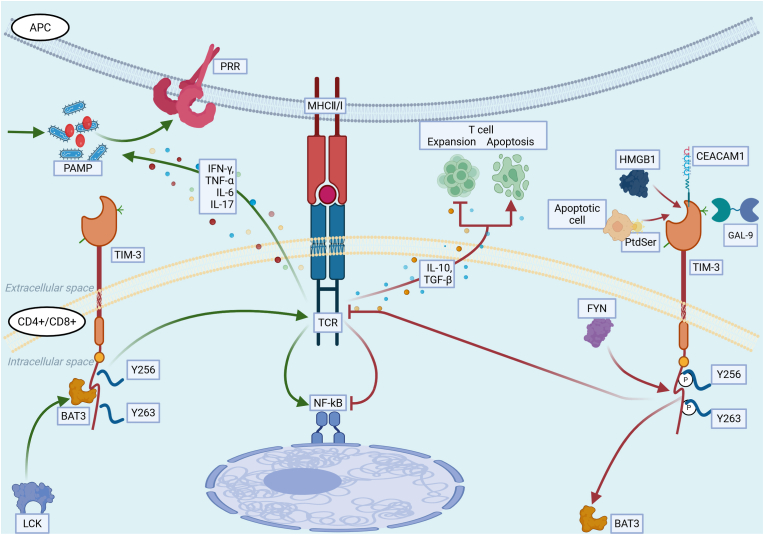
Role and mechanism of TIM-3 in severe infections and sepsis. During early sepsis, APCs recognize PAMPs and transmit signals to T cells through the MHC-TCR. While TIM-3 remains unbound to ligands (green arrow), BAT3 binds to the TIM-3 cytoplasmic tail and recruits phosphorylated lymphocyte-specific tyrosine kinase, enhancing TCR signaling to sustain T cell activation and proinflammatory cytokine release for pathogen clearance. As the disease progresses, TIM-3 is upregulated and binds to Gal-9, HMGB1, PtdSer, and CEACAM-1 (red arrow). This triggers phosphorylation at Y256 and Y263, displacing BAT3 and enabling FYN recruitment to the TIM-3 cytoplasmic tail. Consequently, TIM-3-mediated immunosuppression is activated, which suppresses NF-κB signaling, promotes anti-inflammatory cytokine release, inhibits T cell proliferation, and initiates T cell apoptosis. APC: antigen-presenting cells; PAMP: pathogen-associated molecular pattern; PRR: pattern recognition receptor; MHC II/I: histocompatibility complex II/I; CD4^+^/CD8^+^: CD4/CD8-positive T lymphocyte; TIM-3: T cell immunoglobulin and mucin domain-containing protein 3; LCK: phosphorylated lymphocyte-specific tyrosine kinase; BAT 3: human leukocyte antigen B-associated transcript 3; Y256/Y263: phosphorylation of tyrosine 256/263; TCR: T cell receptor; NF-κB: nuclear factor kappa-B; FYN: protein tyrosine kinase Fyn; Gal-9: galectin-9; HMGB1: high-mobility group Box 1; PtdSer: phosphatidylserine; CEACAM-1: carcinoembryonic antigen-related cell adhesion molecule 1; IFN-γ: interferon-gamma; TNF-α: tumor necrosis factor-alpha; TGF-β: transforming growth factor-β; IL: interleukin.

#### 2.1.1 Interaction and function of TIM-3 with its ligand Gal-9

Gal-9 was the first identified ligand of TIM-3 and binds to TIM-3 to negatively regulate T cell responses, which can prevent hyperactivation of the immune response. This mechanism is critical for modulating hyperinflammatory responses in sepsis and preventing autoimmune disorders.

Gal-9, a tandem-repeat galectin that is widely expressed in lymphocytes and other cell types, plays diverse roles in immune homeostasis, inflammation, malignancies, and autoimmune diseases.^30^ It binds to the glycan structure of the TIM-3 IgV domain and triggers TIM-3 oligomerization and phosphorylation, which ultimately leads to T cell apoptosis. The expression of Gal-9 can be upregulated by IFN-γ, suggesting its involvement in the negative feedback regulation of Th1-mediated inflammatory responses.^17^ The binding between TIM-3 and Gal-9 induces the phosphorylation of intracellular tyrosine residues (Y256 and Y263) on TIM-3, which leads to the dissociation of BAT3 from the TIM-3 cytoplasmic domain and the recruitment of Fyn. Consequently, immune synapse formation is disrupted, resulting in T cell nonfunction or apoptosis.^11^ For example, the TIM-3/Gal-9 interaction inhibits immune synapse formation between T cells and antigen-presenting cells (APCs) and suppresses T cell activation.^31^

Furthermore, Zhu et al.^32^ demonstrated *in vitro* that Gal-9 induces calcium influx, cell aggregation, and death in Th1 cells, whereas *in vivo*, it reduces IFN-γ production in a TIM-3-dependent manner. In the early stage of the immune response, the interaction between TIM-3 and Gal-9 initiates inhibitory signaling, and high Gal-9 expression suppresses excessive Th1 activation, balancing T helper type 1 cells (Th1) and T helper type 2 cells (Th2) responses and shifting immune homeostasis toward an anti-inflammatory state. In chronic viral infections (such as HIV), however, the TIM-3/Gal-9 interaction reduces the susceptibility of activated CD4-positive T lymphocyte (CD4^+^ T) to HIV-1, whereas resting CD4^+^ T cells may exhibit enhanced HIV-1 infection via the PD-1 interaction.^33^ In a *Klebsiella pneumoniae* infection model, TIM-3/Gal-9 binding promoted the apoptosis of Th1 and T helper type 17 cells (Th17), downregulated interleukin (IL)-17, and impaired neutrophil function, exerting immunosuppressive effects.^34^ Interestingly, the TIM-3/Gal-9 axis also maintains normal pregnancy by inhibiting apoptosis and reducing proinflammatory cytokine expression.^27^

In sepsis, Kadowaki et al.^35^ reported that Gal-9 amplifies immune responses by TIM-3-expressing cells (such as natural killer T (NKT) cells and plasmacytoid dendritic cell antigen-1 positive, CD11c positive macrophages, reduces TNF-α, IL-6, IL-10, and HMGB1 levels, increases plasma and splenic IL-15 and IL-17 levels at the same time, and prolongs survival in septic mice without direct antibacterial effects. Wei et al.^36^ found that TIM-3 expression on hepatic CD8-positive T lymphocyte (CD8^+^ T cells) was significantly upregulated in a biphasic manner in septic mice. Competitive inhibition of Gal-9 with α-lactose can suppress TIM-3 activity, reduce TIM-3 expression on CD8^+^ T cells, decrease their degree of apoptosis, and attenuate liver injury. These findings demonstrate that TIM-3 pathway intervention is a viable strategy to attenuate sepsis-induced immunopathology by restoring immune homeostasis.

#### 2.1.2 Interaction and function of TIM-3 with HMGB1

HMGB1 is a highly conserved nuclear protein ubiquitously expressed in eukaryotic cells. In addition to its intracellular role as a chromatin-binding factor, extracellular HMGB1 released from inflammatory and necrotic cells can stimulate monocytes, macrophages, and neutrophils to secrete other inflammatory cytokines, leading to hyperactivation of the inflammatory response.^37^ It can also bind to receptors for advanced glycation end products and trigger the activation of proinflammatory signaling pathways.^38^

Li et al.^39^ demonstrated that regulatory T cells (Tregs) exert a protective effect against acute lung injury by mitigating lipopolysaccharide (LPS)-induced pulmonary inflammation, reducing pro-inflammatory cytokine levels (such as TNF-α and IL-6), and promoting macrophage polarization toward the M2 anti-inflammatory phenotype. However, HMGB1 impairs the immunosuppressive function of Tregs via the toll-like receptor 4 (TLR4) signaling pathway by reducing Treg population, downregulating Forkhead box P3 (FoxP3) expression, and suppressing IL-10/transforming growth factor-β (TGF-β) secretion. Additionally, HMGB1 interferes with the polarization of macrophage M2, ultimately exacerbating LPS-induced acute lung injury.^40^

As a ligand of TIM-3, HMGB1 binds to TIM-3 and attenuates immune cell responses to inflammatory stimuli, suppressing excessive inflammation and maintaining immune homeostasis.^41^ For instance, under stress conditions (e.g., trauma and infection), cells release HMGB1, which binds to TIM-3 and interferes with HMGB1-mediated activation of innate immune responses.^29^ Huang et al.^7^ demonstrated that, in sepsis, HMGB1 binding to TIM-3 suppresses nuclear factor kappa-B (NF-κB) activation in TIM-3^+^ CD4^+^ T cells. This suppression occurs through the inhibition of phosphoinositide 3-kinase (PI3K), protein kinase B (AKT), inhibitor of kappa B alpha, and p65 phosphorylation, which ultimately leads to T cell "exhaustion" and immunosuppression by reducing inflammatory cytokine release and induction.

This regulatory mechanism is particularly crucial during the immunosuppressive phase of sepsis, when the body must both prevent excessive inflammation-induced tissue damage and maintain immune balance to avoid secondary infections. Unfortunately, this immunosuppressed state may impair pathogen clearance, paradoxically increasing susceptibility to secondary infections.

#### 2.1.3 TIM-3 interaction with PtdSer and its functional implications

PtdSer is a negatively charged phospholipid in eukaryotic cell membranes that plays a critical role in cellular signaling. In healthy cells, PtdSer is predominantly localized to the inner leaflet of the plasma membrane; during cell apoptosis, PtdSer is translocated to the outer membrane surface, where it exposes an "eat-me" signal, making its externalization an early hallmark of programmed cell death.^42^

PtdSer is a ligand for TIM-3 that binds to its metal ion-dependent ligand-binding site within the IgV domain. During the initiation phase of apoptosis, PtdSer is redistributed from the inner to the outer plasma membrane, marking the cell for phagocytosis. It is recognized by macrophages and binds to TIM-3 to trigger signaling pathways that enhance apoptotic cell engulfment.^29^ Interestingly, while TIM-3-expressing fibroblasts or APCs can engulf apoptotic targets, TIM-3-positive T cells or B-cells form complexes with dying cells without internalizing them.^43^

The TIM-3-PtdSer interaction modulates NF-κB pathway activity and the expression of IL-2.^17^ It also disrupts the PI3K/mechanistic target of rapamycin complex 1/Phosphorylated Ribosomal Protein S6 signaling axis. As a result, NK cell activity is impaired, activation is suppressed, and cytotoxicity is diminished, which leads to attenuated target cell killing and decreased production of effector cytokines such as IFN-γ and TNF-α.^41^ Surprisingly, during HIV infection, TIM-3 exhibits antiviral activity by binding to the viral envelope PtdSer, inhibiting virion release and transmission.^44^

#### 2.1.4 TIM-3 interaction with CEACAM-1 and its functional consequences

CEACAM-1, a member of the primitive carcinoembryonic antigen gene superfamily, is expressed in leukocytes, epithelial cells, and vascular endothelial cells in both humans and rodents.^45^ CEACAM-1 is not only associated with the early stages of angiogenesis but also regulates the proliferation, migration, and differentiation of murine endothelial cells. Moreover, it participates in modulating vascular remodeling *in vivo*.^46^

The TIM-3-CEACAM-1 interaction plays a pivotal role in immune regulation by modulating intercellular communication and signaling between immune cells, which can influence the intensity and duration of immune responses.^41^ As noted above, CEACAM-1 serves as a TIM-3 ligand. TIM-3 forms a heterodimer with CEACAM-1 in the CC' and FG loops of its IgV-like N-terminal domain.^47^ TIM-3 binding to CEACAM-1 triggers the phosphorylation of Y256 and Y263 in its cytoplasmic tail, causing BAT3 release and sustained inhibition of TCR signaling.^11^ This interaction also enhances TIM-3's inhibitory function by promoting its maturation and surface expression, ultimately impairing T cell activity and inducing Th1 apoptosis.^48^

The interaction between TIM-3 and CEACAM-1 fosters an immunosuppressive microenvironment that is conducive to chronic viral infections. However, this mechanism is essential for maintaining T cell tolerance and regulating autoimmune responses. Blockade of CEACAM-1 and TIM-3 enhances both the number and function of T cells, promotes IFN-γ production, and thereby facilitates pathogen clearance.^47^

Kared et al.^49^ demonstrated that CEACAM-1 suppresses NK-cell function by inhibiting the transcription Factor T-bet, but TIM-3 blockade reverses this suppression; as a result, cytokine secretion and cytotoxic activity are restored. In chronic viral infections (such as HIV), TIM-3 downregulation is correlated with NK-cell exhaustion. Targeting TIM-3 or CEACAM-1 may restore the function of NK cells and offer a novel therapeutic strategy. Conversely, in sepsis, TIM-3/ligand interactions exacerbate immunosuppression and lead to T cell dysfunction.^29^

These evidences indicate that TIM-3's immune regulatory function in sepsis may be ligand-dependent: binding to Gal-9, HMGB1, PtdSer, or CEACAM1 triggers immune suppression, whereas unbound TIM-3 may promote immune activation, rendering it an attractive target for sepsis immunotherapy.

### 2.2 Role and underlying mechanisms of TIM-3 in regulating immune cell function

The expression level of TIM-3 on immune cells can reflect their functional state and immune activity. However, the mechanisms by which TIM-3 regulates these cells may differ (Table 1).

**Table 1:** The effects of TIM-3 on different immune cell types.

Cell types	Effect	Cytokines
T cell	Exhaustion Treg differentiation	IFN-γ, TNF-α, IL-17 ↓ IL-10 ↑
Macrophage	Exhaustion M2 polarization	TNF-α, IL-6 ↓ IL-10, TGF-β ↑
Dendritic cells	Exhaustion	IFN-α, TNF-α, IL-12 ↓
NK cell	Exhaustion	IFN-γ, TNF-α ↓ IL-10 ↑

IFN-γ: interferon-gamma; TNF-α: tumor necrosis factor-alpha; IFN-α: interferon-alpha; TGF-β: transforming growth factor-β; IL: interleukin.

#### 2.2.1 The impact of TIM-3 on T cell function

TIM-3 was initially identified as a surface marker selectively expressed on Th1 cells but not on Th2 cells, making it a key distinguishing feature between these subsets.^13^ TIM-3 plays a critical role in balancing Th1 and Th2 responses. Specifically, its expression on Th1 cells may indirectly regulate Th2 cell activity, and this mechanism is vital for controlling the progression of allergic and autoimmune diseases.^32^

Studies have shown that, in sepsis, upregulated TIM-3 on T cells interacts with Gal-9 to modulate the Th17/Treg balance, mitigating inflammatory responses and tissue damage. The Gal-9/TIM-3 pathway negatively regulates Th1 immunity by inducing apoptosis in Th1 and Th17 cells while promoting the expansion and immunosuppressive function of Tregs. This significantly reduces the Th17 population, decreases proinflammatory IL-17 levels, and elevates anti-inflammatory IL-10 secretion alongside Treg proportions, which collectively attenuate inflammation.^35^^,^^50^

He et al.^51^ demonstrated that in recurrent sepsis, mice exhibited markedly reduced MHC-II expression and impaired APC function. After secondary infection with H1N1, these mice presented increased viral loads in their lung tissue but attenuated pulmonary pathology. This was accompanied by diminished proinflammatory cytokines, elevated IL-10, reduced CD4^+^ T cell infiltration, increased expression of inhibitory receptors (PD-1 and TIM-3), and expanded Treg populations. Consequently, viral clearance is compromised, leading to increased morbidity and mortality during secondary infection.

Moreover, TIM-3 is an immune checkpoint molecule with dual regulatory roles. Its expression and function are modulated by various extracellular signals and transcription factors, and its downstream signaling involves complex molecular interactions. TIM-3 is often associated with detrimental effects in T cell exhaustion; however, it also serves as a critical mechanism to prevent excessive responses to self-antigens.

In immune responses, TIM-3 maintains immune homeostasis by suppressing the hyperactivation of immune cells, including T cells. For example, in autoimmune diseases such as systemic lupus erythematosus, multiple sclerosis, and rheumatoid arthritis, decreased TIM-3 expression is correlated with T cell overactivation, indicating disease progression. In contrast, sustained upregulation of TIM-3 suppresses T cell activity, which is correlated with disease remission. These findings demonstrate the protective role of TIM-3 in preserving immune balance.^17^

However, if antigen stimulation persists, for example, in tumors and chronic infections, including late-stage sepsis, TIM-3 overexpression induces T cell dysfunction, contributing to adverse outcomes such as tumor immune evasion, pathogen immune escape, and immunosuppression.^29^ Consequently, excessive TIM-3 expression has dichotomous effects: on the one hand, it may protect the host from hyperinflammatory damage, mitigating sepsis-induced tissue injury; on the other hand, it can induce immunosuppression in late-stage sepsis, increasing the risk of secondary infections.^52^

#### 2.2.2 The impact of TIM-3 on macrophage function

Multiple studies have demonstrated that macrophages can dynamically switch between the M1 and M2 phenotypes. M1 macrophages secrete proinflammatory factors such as IFN-γ, TNF-α, and reactive oxygen species (ROS), which primarily mediate antimicrobial and antitumor responses. However, excessive M1 activation may also lead to inflammation and tissue damage.^53^ In contrast, M2 macrophages express anti-inflammatory cytokines (including IL-10, TGF-β, and arginase) and promote tissue repair, whereas dysregulated M2 polarization can contribute to aberrant cell proliferation.^54^

Research by Yang et al.^55^ found that during sepsis, toll-like receptors sense pathogens and activate innate immune responses. A critical step involves TLR4 in macrophages recognizing pathogen-associated molecular pattern (PAMPs), particularly LPS; for example, TLR4 binds to LPS and initiates myeloid differentiation primary response 88 (MyD88)-dependent signaling, ultimately activating NF-κB to upregulate proinflammatory cytokines. However, TIM-3 inhibits this process by upregulating A20 (an NF-κB inhibitor) and activating the PI3K-AKT pathway, thereby suppressing LPS-TLR4-mediated NF-κB signaling and reducing the production of the proinflammatory cytokines TNF-α and IL-6. Moreover, increased TIM-3 expression enhances M2 macrophage polarization, further attenuating inflammation and promoting an immunosuppressive microenvironment.^29^

Hou et al.^25^ demonstrated that in early-stage malaria infection, macrophages predominantly exhibit an M1 phenotype, which is characterized by strong phagocytic activity and antiparasitic effects. Moreover, TIM-3's blockade further promoted M1 polarization. However, as infection progresses and becomes chronic, macrophages shift toward the M2 phenotype, which is characterized by increased arginase-1 and IL-10 expression. TIM-3 inhibition suppresses this M2 transition, sustaining the antimalarial activity of macrophages. Similarly, other studies on silicosis confirmed that TIM-3 expression modulates macrophage polarization and phagocytic capacity.^56^

Furthermore, Wang et al.^57^ found that, in *Listeria* infections, TIM-3 suppresses the nuclear factor erythroid 2-related factor 2 (Nrf2) signaling pathway, which can downregulate cluster of differentiation 36 **(**CD36) and heme oxygenase-1 expression, interfere with macrophage phagocytosis and bacterial clearance, and ultimately exacerbate infection. Recently, Zhou et al.^58^ reported that upregulated TIM-3 in macrophages further increases membrane-associated RING-CH-type finger 8 (MARCH8) (a ubiquitin E3 ligase) expression, promoting K48-linked ubiquitination and degradation of MHC-II, which suppresses MHC-II expression, consequently impairing effector CD4^+^ T cell differentiation and facilitating immune evasion of the H1N1 virus.

In summary, TIM-3 inhibits macrophage activation, diminishes cytokine production, and promotes M2 polarization, ultimately impairing phagocytic and bactericidal functions while fostering an immunosuppressive state.

#### 2.2.3 Effects of TIM-3 on DC function

DCs, a type of professional antigen-presenting cell in the immune system, play a pivotal role in bridging innate and adaptive immunity. They regulate the direction and intensity of adaptive immune responses, exerting crucial effects on immune activation, immune tolerance, and immunomodulation through their heterogeneity and diverse functional mechanisms, and they also hold potential therapeutic value for various diseases. Moreover, they can sense microenvironmental changes and accordingly modulate adaptive immune responses, and are capable of inducing either immune activation or tolerance, depending on environmental cues. In infectious diseases, including viral, bacterial, and parasitic infections, DCs contribute to pathogen clearance by activating effector T cells and regulating immune responses.^59^

Multiple studies have demonstrated that TIM-3 is also expressed on DCs, where it exhibits inhibitory effects by suppressing antigen presentation and promoting immune tolerance.^60^ Schwartz et al.^61^ found that elevated TIM-3 expression during HIV infection impaired the capacity of plasmacytoid DCs to produce IFN-α and TNF-α, consequently suppressing plasmacytoid DC function. In addition, the interaction between TIM-3 expressed on DCs and CEACAM-1 may influence the maturation and function of DCs, consequently interfering with antigen presentation and T cell activation.^14^

During sepsis progression, increased TIM-3 expression on DCs facilitates binding with HMGB1, which suppresses TLR-mediated innate immune responses. This interaction impedes DC maturation and antigen-presenting capacity, subsequently impairing T cell activation and differentiation while promoting an immunosuppressive state.^29^

#### 2.2.4 The impact of TIM-3 on NK cells

NK cells, a subset of lymphocytes, play crucial roles in both innate and adaptive immune responses.^62^ As the third major lymphocyte population alongside T cells and B cells, NK cells are essential components of the immune system that induce immune-mediated killing of malignant cells and virions. The main mechanism involves regulating the balance of activating and inhibitory receptors on the cell surface. When activating receptors recognize specific ligands on tumor or virus-infected cells that overwhelm inhibitory signals, NK cells become activated and exert cytotoxic effects by triggering apoptosis in target cells. Additionally, NK cells secrete various cytokines and chemokines (such as TNF-α, IL-10, and IFN-γ) that modulate immune system activity to enhance the functions of other immune cells. Through interactions with antigen-presenting cells such as DCs, NK cells participate in comprehensive immune network activation, synergistically amplifying antitumor and anti-infection responses.^63^ NK cells also perform immune surveillance by continuously patrolling the body to detect and eliminate aberrant cells, preventing tumorigenesis and infection to maintain immune homeostasis.^64^ Notably, NK cells also exhibit long-term immune functions by forming memory-like cells that demonstrate enhanced responsiveness when reactivated.^62^

Emerging evidence indicates that TIM-3 is expressed on NK cells. Wang et al.^65^ demonstrated in tuberculosis studies that patients with active tuberculosis presented impaired NK cell function negatively correlated with elevated TIM-3 expression, which decreased NK cell activation, cytokine secretion, and degranulation potential. The blockade of TIM-3 restored NK cell functionality and improved *Mycobacterium tuberculosis* control. Similarly, during sepsis, upregulated TIM-3 on NK cells mediates functional impairment by suppressing cytotoxicity and antiviral immunity.^66^ Hou et al.^67^ further confirmed in LPS-induced septic mice that the TIM-3-Gal-9 interaction negatively regulates NK cell function, with TIM-3 levels inversely correlated with IFN-γ production. However, TIM-3 inhibition reduced NK apoptosis while increasing IFN-γ secretion and target cell cytotoxicity, indicating the immunosuppressive role of TIM-3 in sepsis.

In contrast, Ndhlovu et al.^68^ reported that in humans, TIM-3 is highly expressed on mature NK cells (similar to Th1 cells) and can be further upregulated by cytokines (IL-12, IL-18, and IL-15). TIM-3^+^ NK cells exhibited superior cytokine production and cytotoxicity when stimulated, suggesting that TIM-3 is a marker of functionally competent subsets rather than exhaustion. However, ligand-bound TIM-3 still exerts inhibitory effects on NK cells. These findings provide critical insights into NK cell immunoregulation and inform potential TIM-3-targeted immunotherapeutic strategies.

### 2.3 TIM-3 synergizes with either PD-1 or other immune checkpoints

T cell exhaustion is characterized by reduced proliferative capacity, diminished cytokine production, impaired cytotoxicity,^69^ and the overexpression of a distinct set of inhibitory receptors, including PD-1, CTLA-4, LAG-3, and TIM-3.^70^ These inhibitory receptors collectively form a complex immunoregulatory network that interacts through distinct mechanisms and signaling pathways to maintain immune system homeostasis.^71^

Researchers have observed that exhausted T cells frequently exhibit co-expression of the inhibitory receptors PD-1 and TIM-3.^72^^,^^73^ This synergistic inhibitory effect plays a significant role in tumor immune evasion and tolerance. In hepatocellular carcinoma, for instance, dual blockade of TIM-3 and PD-1 demonstrates superior efficacy compared to monotherapy by enhancing TNF-α and IFN-γ secretion while reducing IL-10/IL-6 levels, thereby alleviating T cell exhaustion and more effectively suppressing tumor growth.^74^ Studies by Yan et al.^75^ and Kandel et al.^76^ further confirm that this combination therapy synergistically enhances T cell activity and overcomes drug resistance observed with single-agent treatments.

Similar findings have been reported in infectious diseases. During lymphocytic choriomeningitis virus infection, TIM-3 and PD-1 cooperatively regulate CD8^+^ T cell exhaustion, and their co-expression correlates with more severe CD8^+^ T cell dysfunction, while dual blockade of these pathways significantly improves CD8^+^ T cell responses and viral control in chronically infected mice.^77^ Zilber et al.^69^ reached comparable conclusions in their HIV infection study.

Bidar et al.^78^ observed that COVID-19 critically ill patients exhibited markedly reduced proliferative capacity and IFN-γ production in CD4^+^ T cells and CD8^+^ T cells with high expression of PD-1, PD-L1, CTLA-4, and TIM-3. This phenomenon was similarly documented by Boomer et al.^79^ in septic patients. Additionally, Xia et al.^80^ demonstrated that antibody blockade of PD-1 and TIM-3 increased the proportion of IL-10- and TNF-α-expressing monocytes in peripheral blood while elevating serum levels of IL-6, IL-10, and TNF-α, collectively contributing to functional recovery of lymphocytes and monocytes.

Thus, we can conclude that the synergistic interaction between TIM-3 and PD-1, along with other negative immune costimulatory molecules, may play an important immunosuppressive role during sepsis. The combined blockade of TIM-3 and other inhibitory receptors could emerge as a promising therapeutic strategy for sepsis.

### 2.4 TIM-3 exhibits dynamic functional plasticity across disease phases

The biological functions of TIM-3 are complex and multifaceted, with its role varying across different diseases or even different stages of the same disease. Studies suggest that under specific conditions, such as acute infection, TIM-3 exhibits costimulatory activity by enhancing ribosomal protein S6 phosphorylation through the MEK-ERK and PI3K-Akt signaling pathways. This co-stimulation potentiates TCR signaling, promoting T cell activation rather than apoptosis or exhaustion, highlighting the intricate and context-dependent regulatory role of TIM-3 in T cell responses.^81^

In the early phase of sepsis, TIM-3 upregulation may facilitate the activation of NKT cells and the secretion of proinflammatory cytokines to clear pathogens. However, sustained high TIM-3 expression in later disease stages may induce excessive NKT apoptosis and promote immunosuppression, ultimately compromising patient recovery.^82^ Similarly, in COVID-19 patients, TIM-3^+^ NKT cells exhibit increased production of cytokines, including IFN-γ, IL-4, and IL-10, and increased expression of coinhibitory receptors, such as PD-1, CTLA-4, and LAG-3, indicating a state of simultaneous activation and exhaustion.^83^ Notably, in the early stage of parasitic infections, such as malaria, TIM-3 upregulation may promote T cell activation and inflammatory cytokine release to combat pathogens, whereas persistently elevated TIM-3 expression during advanced phases may trigger aberrant apoptotic cascades and foster immunosuppressive states, ultimately impeding physiological recovery.^84^

Gorman et al.^85^ found that TIM-3 expression gradually increases during Th1 cell differentiation, particularly upon secondary TCR stimulation. TIM-3^+^ Th1 cells exhibit enhanced effector functions, including robust production of IFN-γ and TNF. In addition, the role of TIM-3 appears to be stimulus dependent: under acute stimulation, TIM-3^+^ Th1 cells show no increased apoptosis but rather augmented effector activity, whereas chronic stimulation correlates TIM-3 expression with T cell exhaustion. A separate study by Gorman et al.^86^ demonstrated that TIM-3 potentiates CD8^+^ T cell responses during acute *Listeria* infection by increasing proliferation and effector function. Conversely, in chronic infections, TIM-3 is associated with CD8^+^ T cell exhaustion—although it does not directly regulate apoptosis—possibly by suppressing MHC-I expression in macrophages to impair antigen presentation and facilitate immune evasion.^87^

Therefore, the biological functions of TIM-3 may vary depending on the disease stage and type of stimulation, exhibiting immune-enhancing effects during acute infection or transient stimuli while mediating immunosuppression in chronic phases or under sustained stimulation. These findings provide new insights into the complex role of TIM-3 in immune regulation and may have important implications for the development of immunotherapeutic strategies against infections and tumors.

## 3 Clinical significances of TIM-3 in severe infections and sepsis

### 3.1 Potential as a biomarker

Due to its role in mediating immune suppression and tolerance, TIM-3 demonstrates significant potential as a biomarker in various contexts. During pregnancy, elevated TIM-3 expression facilitates the establishment and maintenance of maternal-fetal immune tolerance,^88^ while its downregulation may indicate abnormal pregnancy outcomes.^27^^,^^28^^,^^89^ Conversely, in tumors and infectious diseases, heightened TIM-3 expression usually reflects immune dysfunction and poorer prognosis. Increased TIM-3 levels correlate with functional exhaustion and impairment of critical immune cells, including T lymphocytes, macrophages, and NK cells.

Li et al.^19^ demonstrated that serum TIM-3 and Gal-9 levels, along with the relative decline rate of Gal-9, serve as potential biomarkers for predicting infection risk within the first year after kidney transplantation, enabling identification of high-risk recipients. Similarly, Penatzer et al.^90^ reported TIM-3's strong predictive value for nosocomial infections in pediatric burn patients.

Despite decades of sepsis research, its pathophysiological mechanisms remain incompletely understood, and reliable biomarkers for monitoring sepsis or guiding therapeutic interventions are still lacking.^91^ Existing evidence indicates TIM-3 functions as a negative immune regulator, with elevated expression across various immune cells correlating with the development of immune paralysis and dysfunction in sepsis patients. TIM-3 upregulation may exacerbate immunosuppression by impairing T cell and myeloid cell functionality.^52^

Notably, TIM-3 overexpression is associated with monocyte dysfunction in sepsis patients, potentially increasing susceptibility to secondary infections. Clinical observations reveal a significant correlation between TIM-3 expression levels and patient outcomes, where higher TIM-3 expression predicts more severe immunosuppression and increased mortality.^66^ Yan et al.^92^ further confirmed these findings, showing significantly elevated TIM-3 expression on CD8^+^ T cells in non-survivors compared to survivors, with expression levels positively correlating with shock duration.

In summary, TIM-3 demonstrates a considerable potential as a robust predictive biomarker for diverse pathological conditions, including chronic inflammation, malignancies, autoimmune diseases, sepsis, as well as physiological pregnancy. Its expression patterns show a promising clinical utility for prognostic evaluation across these disease states.

### 3.2 Therapeutic potential as a treatment target

The prognosis and outcome of diseases are closely associated with the host's immune status. As an immune checkpoint, TIM-3 has demonstrated its potential as a therapeutic target in both oncology and inflammation-related fields. Recent studies have shown that TIM-3 inhibitors, either alone or in combination with PD-1 inhibitors, exhibit promising antitumor activity with good tolerability across various malignancies, although further investigation is required to explore the impact of combination therapies on efficacy.^93^ Additionally, researchers are currently planning to initiate a phase I clinical trial to evaluate the safety and efficacy of KK2845 in patients with relapsed or refractory acute myeloid leukemia.^94^

In sepsis, TIM-3 also demonstrates its potential as a therapeutic target. As previously mentioned, during sepsis, immune cells enter a state of hyperactivation due to stress responses. However, excessive immune reactions can threaten host organs and even survival. The body must therefore maintain immune homeostasis to prevent such outcomes, yet this balance is precarious and can easily shift from one extreme to another. Modulation of the TIM-3 signaling pathway may improve immune function by regulating immune cell activity, offering a novel therapeutic strategy for sepsis.

Preclinical studies have shown that the anti-human TIM-3 monoclonal antibody can inhibit TIM-3-mediated signaling, enhancing the immune activity of T cells and monocytes/macrophages. Specifically, it promotes cytokine production by increasing STAT1 phosphorylation and has been shown to reduce viral load and enhance antiviral immune responses in an H1N1 infection model.^95^ Another study using soluble TIM-3-immunoglobulin to block the TIM-3 pathway reveals its dual role in sepsis: in the early phase, it protects the host from excessive inflammatory damage by suppressing macrophage overactivation, while in the late phase, it prevents worsening immunosuppression by inhibiting Th2 cytokine production.^91^ This suggests that modulating the TIM-3 pathway could serve as a potential therapeutic strategy to prevent sepsis-induced immune dysregulation.

**Fig. 2:**
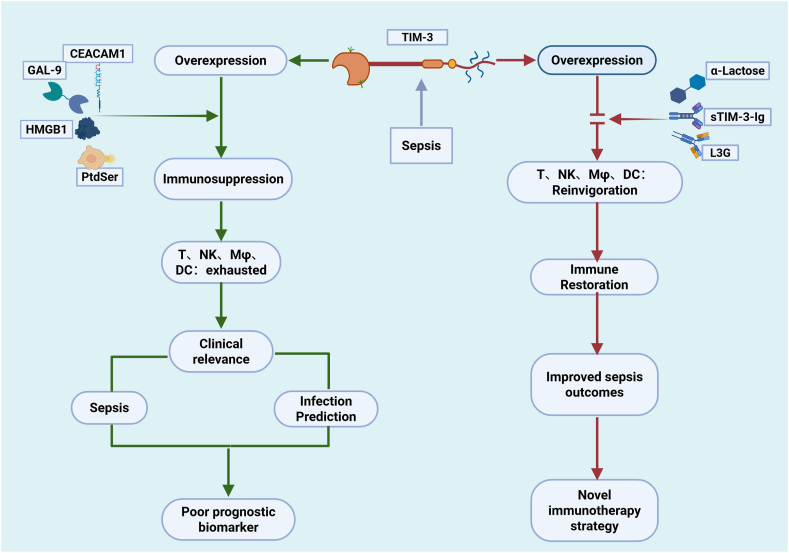
Clinical significance of TIM-3 in severe infections and sepsis. Prior to sepsis, immune cells maintain balanced TIM-3 expression and stable function. Upon sepsis onset, upregulated TIM-3 binds ligands, triggering immunosuppression that exhausts T cells, NK cells, macrophages, and DCs, ultimately impairing host immunity. This leads to more severe immunosuppression and higher mortality, making TIM-3 a prognostic marker for adverse outcomes (green arrow). While the TIM-3-ligand interaction is blocked by monoclonal antibodies, exhausted immune cells partially regain function, restoring patient immunity and improving outcomes. Thus, TIM-3 represents a novel immunotherapeutic target (red arrow). Gal-9: galectin-9; HMGB1: high-mobility group Box 1; PtdSer: phosphatidylserine; CEACAM-1: carcinoembryonic antigen-related cell adhesion molecule 1; T: T cell; DCs: dendritic cells; NK: natural killer cells; Mφ: macrophages; sTIM-3-ig: soluble TIM-3-immunoglobulin; L3G: anti-human TIM-3 monoclonal antibody.

Wei et al.^36^ demonstrated that blocking the TIM-3 pathway with α-lactose reduces CD8^+^ T cell apoptosis in the liver of septic mice, thereby mitigating liver injury. Similarly, Yao et al.^96^ found that α-lactose improves survival in septic mice by inhibiting the TIM-3/Gal-9 pathway, reducing NKT cell apoptosis, and decreasing pro-inflammatory cytokine production. Notably, Huang et al.^7^ showed that both conditional and systemic knockout of TIM-3 in CD4^+^ T cells reduces immunosuppression-related mortality in septic mice. These findings provide a theoretical basis for targeting TIM-3 in sepsis treatment and may offer new clinical therapeutic approaches (Fig. 2).

Although targeting TIM-3 has shown promising results in sepsis treatment research, several critical issues remain to be addressed. First, since septic patients may exhibit different immune statuses at different disease stages, the timing of TIM-3 modulation requires precise determination. Second, the dosage of TIM-3 modulators must be carefully controlled to avoid potential counterproductive effects. Therefore, additional experimental validation is still needed to fully establish the safety and efficacy of TIM-3 as a therapeutic target in sepsis.

## 4 Future research directions

### 4.1 In-depth exploration of TIM-3 signaling pathways

As previously discussed,^71^ TIM-3 has synergistic effects with other negative immune costimulatory molecules in the regulation of immune cells. For example, TIM-3 and PD-1 are frequently co-expressed on severely exhausted T cells. TIM-3 and CTLA-4 primarily suppress T cell activation during the early stages of T cell activation and can further inhibit T cell responses by blocking costimulatory signals. Additionally, the coordinated action of TIM-3 and LAG-3 suppresses T cell proliferation and cytokine secretion. These inhibitory molecules collectively dampen immune cell activation and function through their respective signaling pathways. Therefore, during the immunosuppressive phase of sepsis, a combined immune checkpoint blockade strategy may be necessary to enhance T cell responses, restore immune function, and improve therapeutic outcomes effectively.

### 4.2 Search for new TIM-3 regulation strategies

To date, although significant progress has been made in understanding TIM-3-mediated immune regulation, research on its role in sepsis remains limited. Therefore, future studies should further explore TIM-3-specific signaling pathways and dynamic changes in sepsis to develop targeted therapies. Additionally, investigations should focus on the mechanism of action of TIM-3 in sepsis, including its relationship with inflammatory responses and immune cell function, as well as its interactions with other immune checkpoint proteins (such as PD-1 and CTLA-4) and ligands (including Gal-9 and HMGB1), to assess potential combination therapies. Furthermore, small-molecule inhibitors of TIM-3, along with novel strategies such as gene and cell therapy, should be developed to improve sepsis outcomes. Finally, clinical trials are essential—while TIM-3 is a promising therapeutic target, its broad expression in humans may increase side effects. To validate the prognostic value of TIM-3 genotypes across populations and assess their diagnostic and therapeutic potential, more large-scale animal studies and clinical trials are needed to evaluate the safety of anti-TIM-3 antibodies.

## 5 Conclusion

In summary, TIM-3 plays a complex dual role in sepsis. On the one hand, during sepsis, upregulated TIM-3 on immune cells binds to ligands such as Gal-9 and HMGB1, negatively regulating immune cell function. This suppresses the activation of T cells, macrophages, DCs, and NK cells; promotes their apoptosis or exhaustion; reduces inflammatory cytokine release; and mitigates excessive inflammation, thereby preventing tissue damage. However, excessive TIM-3 expression may induce immunosuppression, increasing the risk of secondary infections. Additionally, TIM-3 synergizes with other immune checkpoint molecules to maintain immune equilibrium. Given its pivotal role in immunomodulation and close association with sepsis prognosis, TIM-3 represents a promising therapeutic target and offers novel insights for treating this life-threatening condition.

Current sepsis management relies primarily on supportive therapies, including antibiotics, fluid resuscitation, and organ support.^66^ However, disrupted immune homeostasis remains a fatal complication.^97^ Although immunotherapies targeting negative costimulatory molecules such as TIM-3 may help restore immune homeostasis in sepsis patients, significant challenges remain. Sepsis-induced immune dysregulation is dynamic and complex, with intricate interactions between cytokines and surface receptors. The safety and efficacy of blocking specific coinhibitory signals require further evaluation. Notable limitations include the following: most current studies employ animal or *in vitro* models with healthy young subjects, whereas clinical sepsis primarily affects elderly or comorbid patients. TIM-3 expression also varies across disease stages and cell types, complicating therapeutic development. Additionally, TIM-3 modulation may cause off-target effects on nonimmune cells. Nevertheless, advances in the understanding of TIM-3 continue to brighten its potential as an effective immunotherapy target for sepsis management.

## CRediT authorship contribution statement

**Shaowen Huang:** Writing – review & editing, Writing – original draft. **Xiaofei Huang:** Visualization. **Xifeng Feng:** Visualization. **Rui Wang:** Visualization. **Fengying Liao:** Visualization. **Di Liu:** Visualization. **Jianhui Sun:** Visualization. **Huacai Zhang:** Visualization. **Anyong Yu:** Writing – review & editing, Conceptualization. **Ling Zeng:** Writing – review & editing, Writing – original draft, Supervision, Project administration, Funding acquisition, Conceptualization.

## Ethical statement

Not applicable.

## Funding

This study was supported by the 10.13039/501100001809National Natural Science Foundation of China (82222038), the Outstanding Young Talents of National Defense Biotechnology (01-SWKJYCJJ06), the Chongqing Outstanding Youth Fund (CSTB2022NSCQ-JQX0017), the Military Clinical Key Specialty Construction Project, and the Special Project for Enhancing Technological Innovation Capacity of 10.13039/501100012397Army Medical University (2023XQN49).

## Declaration of competing interests

All the authors declare that they have no competing financial interests.

## Data Availability

The datasets used for analysis during the current study are available from the corresponding author upon reasonable request.
